# Spontaneous oesophageal perforation secondary to renal colic

**DOI:** 10.1093/omcr/omad112

**Published:** 2023-10-23

**Authors:** Jinghong Zhang, Xinglin Yang, Hui Jiang, Jihai Liu, Jiangshan Wang, Huadong Zhu

**Affiliations:** Central Clinical School, Monash University, Melbourne, VIC, Australia; Department of Internal Medicine, Peking Union Medical College Hospital, Chinese Academy of Medical Sciences & Peking Union Medical College, Beijing, China; Emergency Department, State Key Laboratory of Complex Severe and Rare Diseases, Peking Union Medical College Hospital, Chinese Academy of Medical Sciences & Peking Union Medical College, Beijing, China; Emergency Department, State Key Laboratory of Complex Severe and Rare Diseases, Peking Union Medical College Hospital, Chinese Academy of Medical Sciences & Peking Union Medical College, Beijing, China; Emergency Department, State Key Laboratory of Complex Severe and Rare Diseases, Peking Union Medical College Hospital, Chinese Academy of Medical Sciences & Peking Union Medical College, Beijing, China; Emergency Department, State Key Laboratory of Complex Severe and Rare Diseases, Peking Union Medical College Hospital, Chinese Academy of Medical Sciences & Peking Union Medical College, Beijing, China

## Abstract

Boerhaave syndrome is a rare but potentially life-threatening condition that involves a full-thickness tear of the oesophagus. It accounts for around 15% of all cases of oesophageal perforations and is associated with up to 40% of mortality. Vomiting has been found to be associated with the development of Boerhaave syndrome. However, the aetiology of vomiting varies broadly in the available literatures from alcohol indulgence to marathon running, and from panic attack to radiotherapy for cancer. We present here an unusual case of Boerhaave syndrome where the patient developed spontaneous oesophageal perforation in the setting of renal colic.

## INTRODUCTION

Vomiting is commonly seen in patients presenting to Emergency Department. It could be due to any aetiology, such as myocardial infarction, biliary colic, or renal stones. It is important to identify the cause of vomiting through diagnostic work-up and offer the most appropriate treatment. Nevertheless, emerging evidence suggests that vomiting is associated with the development of Boerhaave syndrome. Boerhaave syndrome is a rare but potentially life-threatening condition that involves a rupture of the oesophagus. Here, we report a case of 65-year-old male who presented to Emergency Department with Boerhaave syndrome resulting from vomiting in the setting of a renal stone.

## CASE REPORT

**Figure 1:**
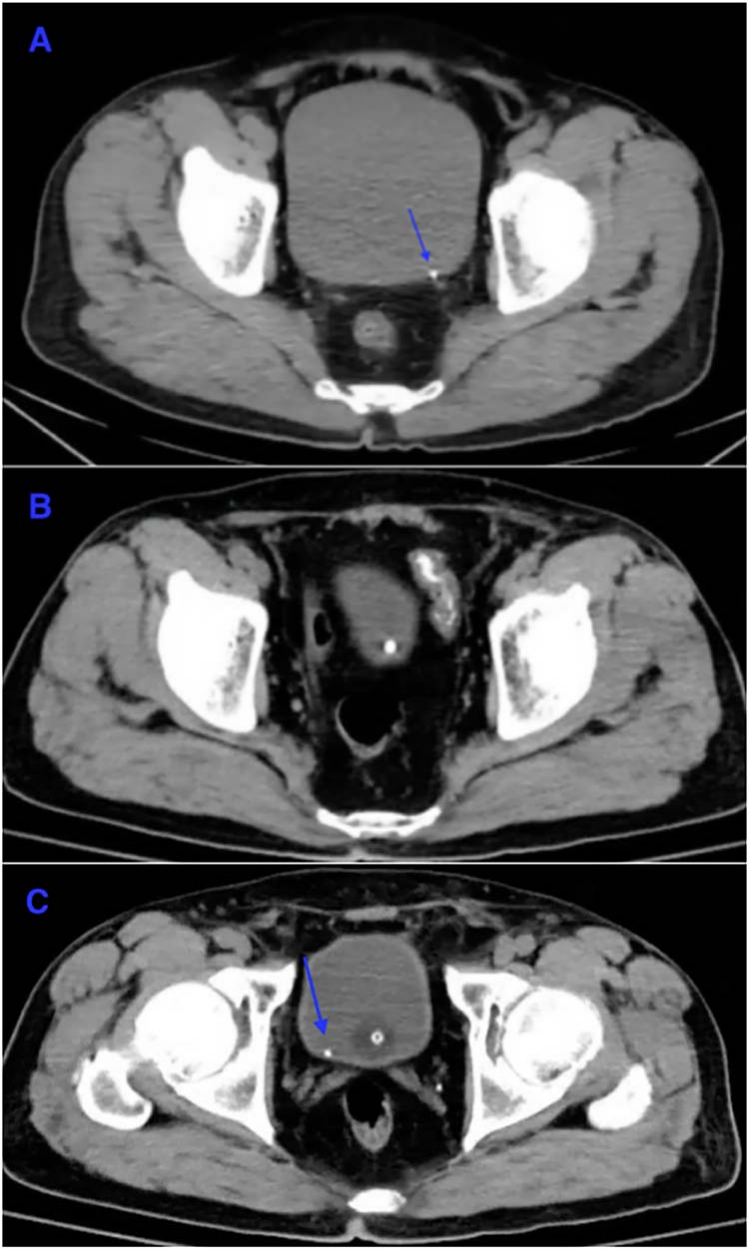
Non-contrast CT scan of the abdomen and pelvis. Axial slice. (**A**) On admission, there was a 4 mm left distal ureteric stone (arrow) is present near vesicoureteric junction. (**B**) On POD10, the left distal ureteric stone has passed. In-dwelling urinary catheter was in situ. (**C**) The left ureteric stone has descended into the bladder (arrow).

A 65-year-old male presented to the Emergency Department of a secondary hospital, which is located in the outskirt of Beijing, with sudden onset of left flank pain radiating to the left scrotum. Patient also had mild nausea and vomiting. Urine dipstick showed the presence of blood (3+). Computer tomography of the kidney, ureter and bladder (CTKUB) revealed a 4 mm left ureteric stone near vesicoureteric junction (Fig. 1A). Patient was otherwise healthy with normal eGFR (>90 ml/min/1.73 m^2^). Patient was determined to be suitable for discharge after his pain had improved with analgesics. On discharge, patient was advised to increase his oral fluid intake and encouraged to mobilize, in order to assist the natural passage of the renal stone.

Over the next 2 h at home, patient had drunk about 4 litres of tea. Although patient felt bloated in the abdomen, he insisted on doing rope skipping in an effort to pass the stone. While jumping, patient suddenly felt nauseous and had a large vomit which appeared coffee ground. Patient also developed severe central chest pain radiating to the back. On his re-presentation to Emergency Department, patient reported feeling increasingly dyspneic. CT of the neck and chest revealed bilateral pleural effusion, dilatation of distal oesophagus, and pneumomediastinum. Oesophageal perforation was highly suspected. Patient was commenced on antibiotics, proton-pump inhibitor, and analgesics. However, patient had increased shortness of breath and continued to feel unwell despite prompt treatment. His oxygen saturation dropped to 87% on room air. Patient was urgently transferred to PUMCH, which is a major tertiary centre in metropolitan Beijing, for emergency surgery and ICU admission.

**Figure 2:**
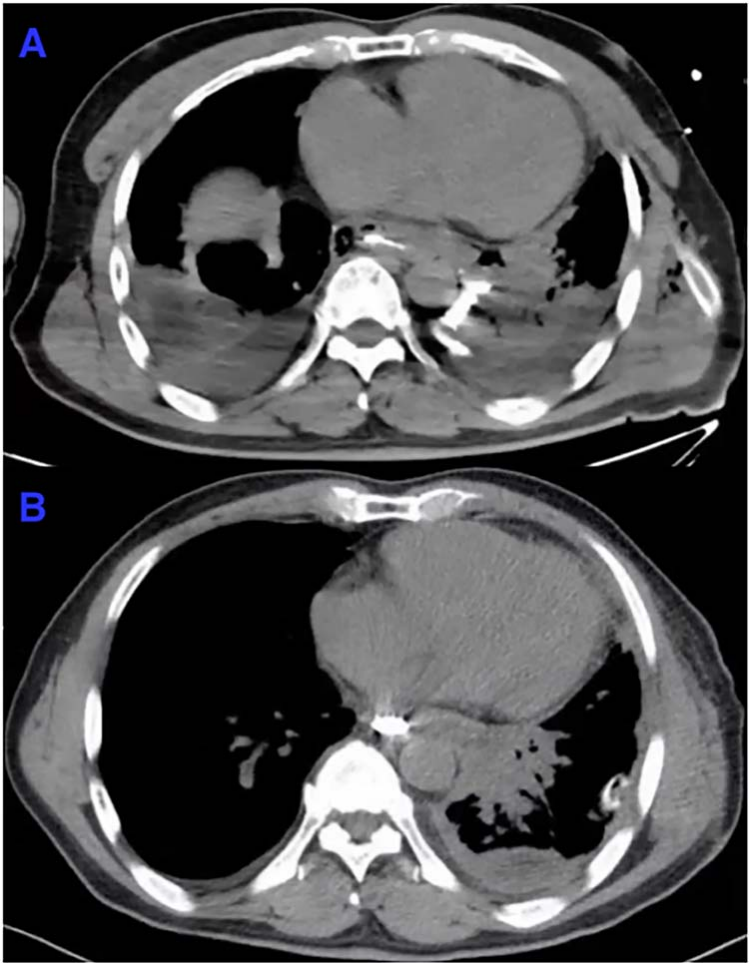
CT oesophagography. Axial slice. (**A**) Horizontal leakage of oral contrast is shown from distal oesophagus to the mediastinum. CT also revealed bilateral pleural effusion on admission. (**B**): On POD10, repeat CT oesophagography did not show leakage of oral contrast. Bilateral pleural effusion, as compared to A, has largely resolved.

On arrival at PUMCH, patient’s vital signs included a pulse rate of 110 bpm, respiratory rate of 24 breaths/min, oxygen saturation of 92% (on 5 l/ min of oxygen), and temperature of 37.0°C. Notable changes in laboratory tests included a pH of 7.31 (normal range: 7.35–7.45), lactate of 3.6 mmol/l (0.5–1), HCO_3_ of 18.9 mmol/l (22–26), CRP of 23.7 mg/l (<5), total bilirubin of 32.6 μmol/l (3–20) and urea of 9.83 mmol/L (1.8–7.1). An intercostal catheter (ICC) was inserted to the left side of the chest wall. 1200 ml of blood-stained fluid appearing in dark red was immediately drained. Subsequent CT oesophagography revealed a distal oesophageal perforation with leakage of oral contrast into the mediastinum and left lower lobe of the lung (Fig. 2A), confirming the diagnosis of oesophageal perforation.

**Figure 3:**
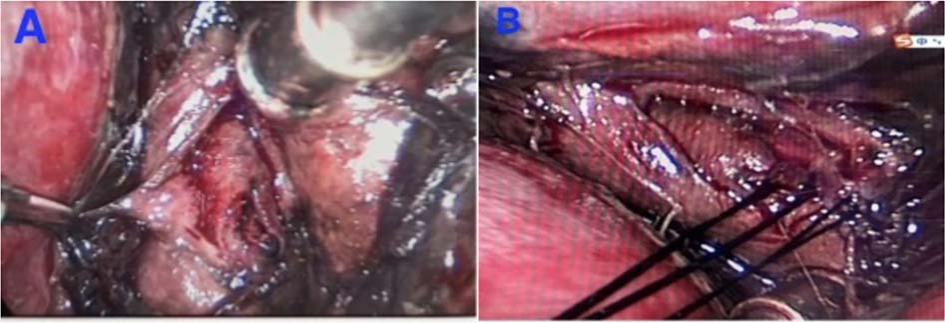
Intraoperative findings during emergency thoracoscopy. (**A**) A rupture (2 cm) of mucosa and a rupture (3 cm) of muscularis in the distal oesophagus. (**B**) Primary repair of the teared mucosa and muscularis layers.

Patient underwent emergency thoracoscopy, debridement, and primary repair of distal oesophageal perforation. Intraoperatively, a longitudinal rupture of mucosa (measuring 2 cm) and another rupture of muscularis (measuring 3 cm) were identified in the distal oesophageal wall near diaphragm (Fig. 3). Copious brownish mediastinal empyema was drained (400 ml) in theatre. The operation was uncomplicated. Patient was commenced on tienam (imipenem/cilastatin) and vancomycin.

Postoperatively, patient was admitted to ICU for haemodynamic monitoring and stabilization. Six hours after surgery, patient’s blood pressure decreased to 84/58 mmHg. Vasopressors were administered for 3 days via CVC. Patient was commenced on total parenteral nutrition (TPN) guided by dietitians. On POD3, tienam and vancomycin were switched to tazocin (piperacillin/tazobactam). On POD5, patient was discharged to ward.

On POD10, repeat CT oesophagography did not reveal any leakage of oral contrast (Fig. 2B). Repeat CTKUB showed that the stone in the distal left ureter had descended into the bladder (Fig. 1B and C). On POD13, patient was discharged from PUMCH and stepped down to a local secondary hospital for wound care and TPN administration. One month later, another CT oesophagography did not show any leakage from the operative site. Patient resumed oral diet 2 months after surgery.

## DISCUSSION

Although Boerhaave syndrome is uncommon, it has been increasingly reported in literature over the last few years [1–8]. Patients with Boerhaave syndrome experienced vomiting in all of the reports prior to their presentations to hospitals. In our case, patient developed Boerhaave syndrome secondary to his vigorous attempt of passing the renal stone after drinking 4 l of fluid, which is unique in mechanism. It is speculated that patient would have avoided the development of Boerhaave syndrome if he did not engage in physical exercise with such high-level intensity.

Boerhaave syndrome in the setting of renal colic has been previously described in 2 case reports [3, 9]. A 30-year-old male, who presented with flank pain, vomiting, and chest pain, was found to have an obstructing renal stone. Patient had pneumomediastinum on imaging. Fortunately, gastrograffin swallow study revealed normal results without any leakage of contrast. Patient ended up receiving primary treatment for the urological stone while his oesophageal perforation was managed conservatively [9]. In the second case, however, a 38-year-old male’s Boerhaave syndrome was initially missed by the treating clinician. The diagnostic focus was only on his renal stone. Pneumomediastinum was incidentally found on CT by radiologist. A subsequent gastrograffin study showed leakage into mediastinum, confirming the diagnosis of Boerhaave syndrome. Patient underwent emergency thoracotomy and repair of the oesophagus [3].

In addition to the surgical repair of the oesophagus, emerging evidence has suggested that endoluminal vacuum (EndoVac) therapy may be an alternative approach in managing oesophageal perforation. EndoVac therapy utilizes a vacuum sponge which is placed within or adjacent to the perforated site through an endoscope [10]. This minimally invasive approach has been shown to be associated with reduced likelihood of postoperative sepsis and mortality in patients with Boerhaave syndrome [10]. Future studies, namely randomized controlled trials, will be needed to evaluate the selection of the most appropriate management taking in account patient comorbidities, pathological results, and imaging findings.

The diagnosis of Boerhaave syndrome can be challenging in Emergency settings as it could be distracted by other acute conditions. Patients, who are otherwise healthy, can suffer from spontaneous oesophageal perforation secondary to vomiting. While the life-threatening diseases (e.g. myocardial infarction and bowel perforation) cannot be missed when patients present with acute pain and vomiting, Boerhaave syndrome should be considered in order to offer timely treatment and reduce mortality rate.

## Data Availability

Some or all datasets generated during and/or analysed during the current study are not publicly available but are available from the corresponding author on reasonable request.

## PRIOR PRESENTATION

No.
